# Small, odd and old: The mysterious *Tarsius pumilus* is the most basal Sulawesi tarsier

**DOI:** 10.1098/rsbl.2021.0642

**Published:** 2022-03-30

**Authors:** Laura Hagemann, Nanda Grow, Yvonne E.-M. B. Bohr, Dyah Perwitasari-Farajallah, Yulius Duma, Sharon L. Gursky, Stefan Merker

**Affiliations:** ^1^ Department of Zoology, State Museum of Natural History Stuttgart, 70191 Stuttgart, Germany; ^2^ Department of Anthropology, Washington State University, Pullman, WA 99164‐4910, USA; ^3^ Institute of Ecology, Evolution and Diversity, Johann Wolfgang Goethe-Universität Frankfurt, 60438 Frankfurt am Main, Germany; ^4^ Department of Biology, Universität Hamburg, 20146 Hamburg, Germany; ^5^ Primate Research Center, IPB University, Bogor 16151, Indonesia; ^6^ Department of Biology, Faculty of Mathematics and Natural Sciences, IPB University, Bogor 16151, Indonesia; ^7^ Faculty of Animal Husbandry and Fisheries, Universitas Tadulako Palu, 94148, Palu, Central Sulawesi, Indonesia; ^8^ Department of Anthropology, Texas A&M University, College Station, TX 77843‐4352, USA

**Keywords:** primates, divergence time, phylogeny, starbeast, biogeography, genetics

## Abstract

In this study, we present the first genetic evidence of the phylogenetic position of *Tarsius pumilus,* the mountain tarsier of Sulawesi, Indonesia*.* This mysterious primate is the only Eastern tarsier species that occurs exclusively in cloud forests above 1800 m.a.s.l. It exhibits striking morphological peculiarities—most prominently its extremely reduced body size, which led to the common name of ‘pygmy tarsier’. However, our results indicate that *T. pumilus* is not an aberrant form of a lowland tarsier, but in fact, the most basal of all Sulawesi tarsiers. Applying a Bayesian multi-locus coalescent approach, we dated the divergence between the *T. pumilus* lineage and the ancestor of all other extant Sulawesi tarsiers to 9.88 Mya. This is as deep as the split between the two other tarsier genera *Carlito* (Philippine tarsiers) and *Cephalopachus* (Western tarsiers), and predates further tarsier diversification on Sulawesi by around 7 Myr. The date coincides with the deepening of the marine environment between eastern and western Sulawesi, which likely led to allopatric speciation between *T. pumilus* or its predecessor in the west and the ancestor of all other Sulawesi tarsiers in the east. As the split preceded the emergence of permanent mountains in western Sulawesi, it is unlikely that the shift to montane habitat has driven the formation of the *T. pumilus* lineage.

## 1. Introduction

The Southeast Asian region Wallacea is home to a spectacular diversity of wildlife characterized by a very high degree of endemism [1]. This is the result of relatively recent large-scale geological changes due to its location in the collision zone between major tectonic plates [2]. As Wallacea's largest island, Sulawesi lies at the heart of these geological processes, and remarkable patterns of allopatric diversity reflect the geological past [3,4]. Approximately 45 million years ago (Mya) the rapid northward movement of Australia caused a restructuring of the Wallacea region, including the split of East Borneo and West Sulawesi that led to the formation of the Makassar Strait [2]. Since the mid-Eocene, the Makassar Strait forms a substantial barrier of deep water between Sundaland and the areas to the east. Most extant terrestrial animal species can exclusively be found on either one or the other side of this barrier, called Wallace's line, although there are some instances of gene flow across the divide [5]. Tarsiers (family Tarsiidae) represent one of the few vertebrate taxa that occur on both sides of Wallace's line, with the Philippine tarsier (*Carlito syrichta*) inhabiting the Philippines, the Western tarsier (*Cephalopachus bancanus*) occurring in parts of Sumatra, Borneo, Bangka, Belitung, the South Natuna islands and the Karimata islands, and Eastern tarsiers (*Tarsius* spp.) inhabiting Sulawesi and the surrounding islands [6].

Tarsiers are small, nocturnal primates whose phylogenetic position within the primate tree has long sparked a major debate. It is now commonly accepted that tarsiers represent the oldest lineage of haplorhine primates, thus forming a sister-clade to Anthropoidea [7]. Sulawesi tarsiers are the most species-rich group, with 12 species currently recognized and likely more to be described [8,9]. Their evolutionary history is closely linked to Sulawesi's complex geological past. Driven by advances in methodology and sampling coverage, our understanding of the tarsiers' evolutionary history has greatly increased over the past decade [10–13].

Multi-locus genetic analyses suggest that during the early Miocene tarsiers dispersed from Proto-Java and arrived in the southern part of Sulawesi. During the Plio-Pleistocene, they expanded northward in consecutive waves, leading to the formation of two lineages, both of which underwent further diversification [10]. This scenario holds a gap of at least 13 Myr between their initial colonization and diversification [10]. However, one significant piece of the puzzle is missing from this prior analyses: the highly elusive *Tarsius pumilus*.

Remarkably little is known about the pygmy or mountain tarsier, *T. pumilus*, deeming it ‘one of the most mysterious primates of the world’ [14]. For decades, the species was known only from two museum specimens collected in 1917 in Central Sulawesi and in 1930 in South Sulawesi [15,16], until in the year 2000, a third individual was accidentally trapped at 2200 m.a.s.l. on Mount Rore Katimbu in Lore Lindu National Park, Central Sulawesi [17]. In 2008, researchers were finally able to locate a group of live mountain tarsiers in the same location, providing the first behavioural observations [18]. In contrast with all other tarsiers, the mountain tarsier exclusively inhabits cloud forest at elevations above 1800 m.a.s.l.[16,19]. *T. pumilus* exhibits morphological and behavioural peculiarities in respect to other extant tarsiers, like elongated nails, evidently reduced scent marking and the lack of a conspicuous duet song in the human hearing range [14,16,18,20]. The most striking characteristic is its reduced body size. With linear measurements about 75% of those seen in other Sulawesi tarsiers [14,16] and an average weight of 55 g [21], *T. pumilus* is the smallest of all extant tarsiers (electronic supplementary material, table S1 and figure S1). Whether these traits are ancestral or adaptations to the mountain habitat remains largely unresolved for now [14,16,18,20].

An outstanding question concerning *T. pumilus* is its evolutionary relationship to other tarsiers, which may help explain the unique traits found in the species. There are three phylogenetic hypotheses about the evolutionary placement of *T. pumilus* [14]. One possibility is that the mountain tarsier is ‘an aberrant lowland tarsier’ that clusters within the known lineages of Sulawesi lowland tarsiers. Another option is that *T. pumilus* is the most basal of all Eastern tarsiers. Finally, the mountain tarsier could be the sister group to all other tarsiers, including the genera *Cephalopachus* and *Carlito*.

In this study, we present the first genetic data on *T. pumilus*. We aim to disentangle competing explanations on the origin of this species and clarify its place in the tarsier phylogeny. By dating divergence events within the Tarsiidae, we link major geological events with tarsier evolutionary history.

## 2. Material and methods

### (a) Dataset

**Figure 1.**
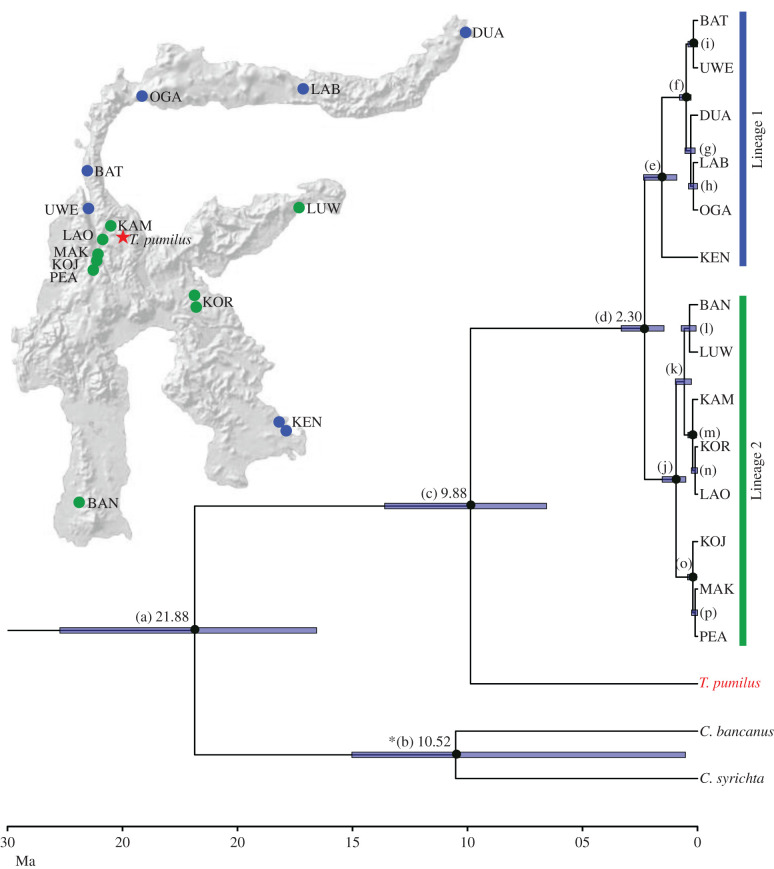
Maximum clade credibility species tree based on five autosomal loci (ABCA1, ADORA3, AXIN1, RAG1 and TTR), outgroups not shown. Numbers represent median node ages in million years. Blue bars represent the 95% highest posterior density (HPD) interval. Nodes with a posterior probability of 0.9 or higher are indicated by a black dot. Branch length is scaled by time. In the upper left corner is a map of Sulawesi, with blue dots representing sampling locations of individuals belonging to Lineage 1, green dots representing those belonging to Lineage 2, and a red star representing *T. pumilus* (modified from [22]). * The width of the HPD is likely a result of the high number (*n* = 28) of variant positions within the RAG1 sequences of *C. syrichta* obtained from NCBI (allelic variant 1: KP642405, allelic variant 2: KP642406). The three‐letter codes denote sampling locations (see text and [10]). For definitions of labels (*a*–*o*), see table 1.

We used publicly available partial sequences of five autosomal genes (intronic: ABCA1 and TTR, exonic: ADORA3, AXIN1 and RAG1, GenBank accession numbers KP642169–KP642408 and KP642434–KP642493) derived from 28 Sulawesi tarsiers, sampled at 14 locations distributed across the island plus corresponding sequences from *C. syrichta* and *C. bancanus* (for details see [10]). We complemented the existing dataset with sequences from four *T. pumilus* individuals sampled in 2012 by N.G. on Mount Rore Katimbu in Lore Lindu National Park (S01° 18′ 37.8″ E120° 18′ 35.5″, figure 1). Individuals were caught with mist nets set up between dusk and dawn in the forest undergrowth (see [21] for details).

At the Primate Research Center PSSP-IPB in Bogor, Indonesia, we used the DNeasy Blood and Tissue Kit (Qiagen) to extract DNA from blood samples (Qiagen FTA spot cards) of 12 individuals and conducted whole-genome amplifications (WGA) using a REPLI-g Mini Kit (Qiagen), before exporting WGA products to our laboratory in Germany.

### (b) Sequencing autosomal loci

The segments were amplified using the HotStarTaq plus PCR Master Mix Kit (Qiagen), filling 10 µl HotStarTaq plus Master Mix (2×), 0.33 mM forward primer, 0.33 mM reverse primer, 200 ng template DNA up with RNase-free water to a total volume of 20 µl. Primer and PCR conditions (electronic supplementary material, table S2) were adapted and slightly modified from Driller *et al*. [10]; a negative control was carried out together with each PCR run. Sequencing of both strands was conducted by LGC Genomics, Berlin, on an ABI 3730xl DNA analyzer. The resulting sequences were manually edited and screened using the software Geneious R10 (https://www.geneious.com) and BioEdit v. 7.2 [23]. Allelic variants were phased using customized nested primers (electronic supplementary material, S1 and table S3).

### (c) Y-haplotype network

In addition to the five autosomal loci, we generated partial sequences of the SRY (sex-determining region of the Y chromosome) locus (electronic supplementary material, table S2). We complemented the sequences of three male *T. pumilus* with available sequences of 59 male Sulawesi tarsiers plus one *C. syrichta* generated in former studies ([4,10,24], Genbank accession numbers KP642409–KP642433, FJ614510–13, FJ614517-21, FJ614523–34, FJ614562–68, HM115985–91). We identified 12 unique Y-haplotypes and used them to construct a TCS network with POPART v. 1.7 [25,26]. Owing to the potentially misleading phylogenetic signal of mtDNA in Sulawesi tarsiers [12], we did not include mitochondrial markers.

### (d) Divergence time estimates

Divergence time estimates were conducted with StarBEAST2 using a Bayesian multi-locus coalescent approach [27,28]. We used sampling locations as traits instead of species, because the species was not defined for all individuals. We fitted a birth–death model [29] with an uncorrelated relaxed lognormal clock [30] based on seven node calibrations suggested by Perelman *et al*. [31] (for details see electronic supplementary material, S2, S3 and table S4).

## 3. Results

### (a) Multi-locus species tree and divergence time

Our Bayesian tree inference reflects the previously held notion that Sulawesi tarsiers form a monophyletic group, with lowland tarsiers having split into two major lineages in the early Pleistocene [10]. Lineage 1 comprises *Tarsius wallacei* (UWE, BAT, figure 1), *T. supriatnai* (OGA, LAB), *T. spectrumgurskyae* (DUA) and *T.* sp. from Kendari (KEN), while Lineage 2 consists of *T. lariang* (PEA, KOJ, MAK), *T. fuscus* (BAN) and *T. dentatus* (LAO, KAM, KOR, LUW). The position of *T. pumilus* was unknown to date. Based on four individuals and five autosomal markers, our analyses suggest that *T. pumilus* is the sister group to all other extant Sulawesi tarsiers and thus represents the most basal Sulawesi tarsier. Dated to 9.88 Mya (median node age, 95% highest posterior density (HPD) 6.57–13.61 Mya), the divergence between lineages leading either to *T. pumilus* or to the other extant species predates the split into the two previously known major lineages of Sulawesi tarsiers (median node age 2.30 Mya, 95% HPD 1.46–3.32 Mya) and subsequent diversification into the other known species by about 7 Myr (figure 1 and table 1).

**Table 1.** Node ages and posterior probabilities. Node labels correspond to node labels in figure 1; splits within Lineage 1 are in italics, splits within Lineage 2 are in bold and the origin of the *Tarsius pumilus* lineage is in bold italics.

node label	node age	lower 95% HPD	upper 95% HPD	posterior probability
a	21.88	16.57	27.74	1.00
b	10.52	0.53	15.04	1.00
***c***	***9.88***	***6.57***	***13.61***	***1.00***
d	2.30	1.46	3.32	1.00
*e*	*1.55*	*0.9*	*2.35*	*0.97*
*f*	*0.50*	*0.28*	*0.78*	*1.00*
*g*	*0.3*	*0.11*	*0.55*	*0.84*
*h*	*0.18*	*0.00*	*0.39*	*0.51*
*i*	*0.18*	*0.00*	*0.41*	*0.91*
**j**	**0.94**	**0.51**	**1.54**	**1.00**
**k**	**0.58**	**0.27**	**0.96**	**0.81**
**l**	**0.35**	**0.06**	**0.71**	**0.37**
**m**	**0.21**	**0.05**	**0.42**	**0.92**
**n**	**0.10**	**0.00**	**0.27**	**0.39**
**o**	**0.20**	**0.04**	**0.43**	**1.00**
**p**	**0.10**	**0.00**	**0.26**	**0.42**

### (b) Y-haplotype network

**Figure 2.**
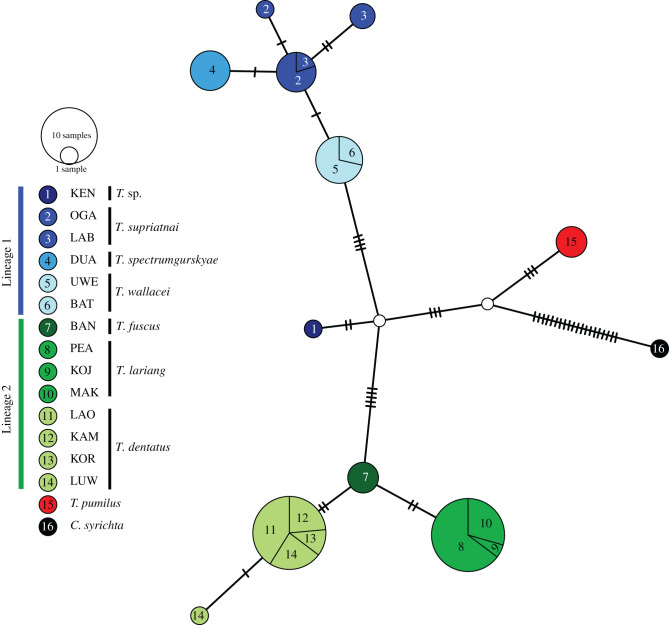
TCS network of 12 SRY haplotypes derived from 63 tarsiers from 16 sampling locations. The area of the circle is proportional to the number of haplotypes. Base pair differences are depicted by slashes.

We inferred 12 SRY haplotypes from 63 male tarsiers; 11 of these haplotypes were already described in Driller *et al*. [10], while the twelfth belongs to *T. pumilus*. Haplotypes are not necessarily unique to single sampling locations, but they are not shared across species. The TCS network shows clustering, here defined by a low amount of sequence differences, of haplotypes within the two previously established lineages, with the male from Kendari being most distinct (figure 2). The three male *T. pumilus* individuals share the same haplotype and cluster with neither of the known lineages. The haplotype with the fewest differences from *T. pumilus* is *Tarsius* sp. *f*rom Kendari, with eight base pair differences, followed by *T. wallacei* with 10 differences and *T. fuscus* with 11 differences.

## 4. Discussion

Based on five autosomal markers and one Y-chromosomal marker, our results unambiguously show that *Tarsius pumilus* is the most basal extant Sulawesi tarsier. Divergence time estimates reveal that the split between *T. pumilus* and the other Sulawesi tarsiers is equally deep as the split between *C. syrichta* and *C. bancanus*, *w*hich are considered to belong to different, monotypic genera [6]. Thus, the mountain tarsier is not merely a lowland tarsier that shifted to the mountains, but the representative of a deep lineage that has experienced roughly 10 Mya of independent evolution. As the diversification of known Sulawesi lowland tarsiers started as late as around 2.3 Mya, and most tarsier species originate less than 1 Mya, *T. pumilus* is exceptional and a keystone in understanding tarsier morphology, behaviour, sociality and distribution.

Reconstructions of the geological past of Sulawesi are continuously refined, but paleogeographic maps become less certain with the depth of time [2,32–34]. The same is true for molecular dating. For example, the estimated divergence date of crown primates differs by more than 25 Myr across studies [31,35,36]. The choice of calibration points strongly influences the outcome of divergence time estimates; the split of *T. pumilus* varies by 2 Myr using the same dataset and method but different calibration points (electronic supplementary material, figure S2 and table S5). Thus, phylogeographic reconstruction inevitably comprises uncertainty, but nevertheless the concurrence between genetic divergence dating and the timing of major geographic events allows for educated guesses on likely scenarios.

Tarsiers likely colonized Sulawesi in the early Miocene (around 20 Mya [10]). At that time, the Sulawesi region was profoundly transformed by the collision of the Sula Spur, a promontory of Australia, with the Southeast Asian margin. This led to the emplacement of ophiolites, the formation of mountains and the rise of landmasses above sea level, especially in East Sulawesi [33]. By 20 Mya, some parts of western and eastern Sulawesi were above sea level. Between 20 and 10 Mya, they were surrounded by a shallow marine environment, and it is possible that a subaerial connection was established at times [33,34]. Subsequently, the region experienced subduction, magmatism and rifting, leading to the uplift and emergence of landmasses but also to the formation of deep water environments that covered central Sulawesi. Thus, from around 10 to 8 Mya onwards, landmasses that now form eastern and western Sulawesi were separated by deep water that likely hindered dispersal [33,34]. Considering the temporal overlap, it is reasonable to infer that the deepening of the marine environment reinforced the barrier to gene flow among tarsiers. The geographic barrier likely led to allopatric speciation, with *T. pumilus* or its predecessor in the west and the ancestor of the remaining extant Sulawesi tarsier species in the east. As late as 3 Mya, a land bridge is thought to have connected east and west Sulawesi [33]. In the late Pliocene and early Pleistocene, Sulawesi experienced a large-scale uplift, resulting in a substantial increase in landmass that enabled species range expansion [37].

Interestingly, the origin of the lineage leading to *T. pumilus* predates the emergence of permanent mountains in western Sulawesi by about 4 Myr [33]. Body size variation related to altitudinal adaption is thus unlikely to have driven tarsier lineage divergence. It remains unclear whether (and if so, when) the small body size of mountain tarsiers evolved in response to living in high-altitude forest or whether being small simply represents the primitive state. Estimated body weights of Eocene and Miocene fossil tarsiers vary greatly [38] but uncertain relationships between fossil and extant species obscure the polarity of character states.

Our findings lift the veil of mystery surrounding *T. pumilus*' phylogenetic position, allowing for a more comprehensive understanding of the biogeography of Wallacea, tarsier phylogeography and character evolution. Broader sampling is needed to infer the current distributional range of *T. pumilus* and to verify our proposed colonization and speciation scenario. Larger-scale sequencing will enable the identification of genes under selection in mountain habitat, in order to distinguish between adaptive and primitive characters.

## Data Availability

The nucleotide sequences generated in this study are accessible at GenBank with the accession numbers OM885831-OM885873. The multi-species alignments in fasta format and the alignments partitioned by codon position (for protein-coding sequences) in nexus format are stored in the Dryad repository and publicly available at https://doi.org/10.5061/dryad.xgxd254j9. These alignments were the basis for the inference of phylogenetic trees, divergence times and the haplotype network as described in detail in the main text and the electronic supplementary material [39].
